# The Implication of Mechanistic Approaches and the Role of the Microbiome in Polycystic Ovary Syndrome (PCOS): A Review

**DOI:** 10.3390/metabo13010129

**Published:** 2023-01-14

**Authors:** Anirban Goutam Mukherjee, Uddesh Ramesh Wanjari, Sandra Kannampuzha, Reshma Murali, Arunraj Namachivayam, Raja Ganesan, Abhijit Dey, Achsha Babu, Kaviyarasi Renu, Balachandar Vellingiri, Gnanasambandan Ramanathan, George Priya Doss C., Nehal Elsherbiny, Amira M. Elsherbini, Alsamman M. Alsamman, Hatem Zayed, Abilash Valsala Gopalakrishnan

**Affiliations:** 1 Department of Biomedical Sciences, School of Biosciences and Technology, Vellore Institute of Technology (VIT), Vellore 632014, India; 2 Institute for Liver and Digestive Diseases, Hallym University, Chuncheon 24252, Republic of Korea; 3 Department of Life Sciences, Presidency University, Kolkata 700073, India; 4 Centre of Molecular Medicine and Diagnostics (COMManD), Department of Biochemistry, Saveetha Dental College and Hospitals, Saveetha Institute of Medical and Technical Sciences, Saveetha University, Chennai 600077, India; 5 Stem Cell and Regenerative Medicine/Translational Research, Department of Zoology, School of Basic Sciences, Central University of Punjab (CUPB), Bathinda 151401, India; 6 Department of Integrative Biology, School of Biosciences and Technology, Vellore Institute of Technology (VIT), Vellore 632014, India; 7 Department of Pharmaceutical Chemistry, Faculty of Pharmacy, University of Tabuk, Tabuk 71491, Saudi Arabia; 8 Department of Biochemistry, Faculty of Pharmacy, Mansoura University, Mansoura 35516, Egypt; 9 Department of Oral Biology, Faculty of Dentistry, Mansoura University, Mansoura 35516, Egypt; 10 Department of Genome Mapping, Molecular Genetics and Genome Mapping Laboratory, Agricultural Genetic Engineering Research Institute, Giza 12619, Egypt; 11 Department of Biomedical Sciences, College of Health Sciences, QU Health, Qatar University, Doha P.O. Box 2713, Qatar

**Keywords:** PCOS, metabolomics, metagenomics, microbiome, therapy

## Abstract

As a complex endocrine and metabolic condition, polycystic ovarian syndrome (PCOS) affects women’s reproductive health. These common symptoms include hirsutism, hyperandrogenism, ovulatory dysfunction, irregular menstruation, and infertility. No one knows what causes it or how to stop it yet. Alterations in gut microbiota composition and disruptions in secondary bile acid production appear to play a causative role in developing PCOS. PCOS pathophysiology and phenotypes are tightly related to both enteric and vaginal bacteria. Patients with PCOS exhibit changed microbiome compositions and decreased microbial diversity. Intestinal microorganisms also alter PCOS patient phenotypes by upregulating or downregulating hormone release, gut-brain mediators, and metabolite synthesis. The human body’s gut microbiota, also known as the “second genome,” can interact with the environment to improve metabolic and immunological function. Inflammation is connected to PCOS and may be caused by dysbiosis in the gut microbiome. This review sheds light on the recently discovered connections between gut microbiota and insulin resistance (IR) and the potential mechanisms of PCOS. This study also describes metabolomic studies to obtain a clear view of PCOS and ways to tackle it.

## 1. Introduction

Polycystic ovary syndrome (PCOS), the most common cause of female endocrine infertility, is characterized by increased ovarian androgen biosynthesis, anovulation, and, as previously stated, infertility [1,2]. However, PCOS has long-term consequences that extend far beyond reproductive age and affect a woman’s overall health [3,4,5]. The prevalence of PCOS ranges from 8% to 13%, depending on the population studied and the diagnostic criteria used [6,7]. Hyperandrogenism (HA) is a fundamental primary disorder of PCOS. The gonadal axis changes the formations of androgenic alopecia, acne, and hirsutism [8]. In 1992, the National Institutes of Health (NIH) used two characteristics to define PCOS: the first is the chronic anovulation stage, and the second one is biochemical hyperandrogenism [9]. Polycystic ovarian morphology (PCOM) has 12 or more follicles measuring from 2 to 9 mm in diameter or an ovarian volume of at least 10 cm^3^. Rotterdam stated it in the meeting of 2003 [10,11,12]. Although the precise cause of PCOS is unknown, several factors have been identified as contributing to the hormonal and metabolic imbalance that can lead to the onset of this syndrome [13]. One of the criteria for determining whether an adult woman has PCOS is the presence of PCOM [2]. As a result of the ovaries reaching their maximum volume and follicle count in the second decade of life, there is difficulty in interpreting and classifying ovarian morphology during this period. It has led to a need for different diagnostic criteria for PCOM during adolescence [14]. According to some reports, the prevalence of PCOM varies with age, being highest in adolescents [15,16,17]. The study by Paulina et al., 2017 found that adolescents with PCOM had higher AMH levels, lower FSH levels, and an absence of HA [18]. The RC criteria, including an assessment of the number of follicles, showed the best association with AMH levels. The two other classifications, based on ovarian volume alone, had similar AUCs. PCOM has historically been associated with elevated AMH levels in healthy adolescents and adults [19,20,21].

The NIH consensus panel suggested the phenotypic approach to classifying PCOS in 2012 [22]. Phenotype A (full-blown syndrome PCOS: HA + OD + PCO) includes HA (clinical or biochemical), ovulatory dysfunction (OD), and polycystic ovaries (PCO) (HA + OD + PCO). Phenotype B (non-PCO PCOS: HA + OD) includes HA and OD. Phenotype C (ovulatory PCOS: HA + PCO) includes HA and PCO. Phenotype D (non-hyperandrogenic PCOS: OD + PCO) includes OD and PCO [23]. According to a study by Polak et al., in phenotype A, higher levels of the android/gynoid ratio (A/G ratio) and visceral adipose tissue (VAT) mass were compared to the control group [24].

## 2. PCOS

### Transgenerational Transmission of PCOS and Its Pathophysiology

The research portrays that PCOS has a 5-fold risk that daughters born with PCOS mothers will inherit the syndrome [25,26]. Infant girls born to PCOS mothers have a longer anogenital distance (AGD), and their daughters have a higher metabolic and androgenic risk [26,27]. Maternal testosterone levels predicted infant AGD in PCOS-afflicted women [28]. Although serum anti-Müllerian hormone (AMH) may play a role, the precise mechanism by which the daughters are exposed to HA is unknown [29]. The mechanism was thought to function by increasing aromatase activity in the placenta via the effect of AMH. Women with PCOS have been shown to have high AMH levels in the second and third trimesters [30,31]. Further research is required to understand how AMH influences human transgenerational transmission entirely. 

In humans, the follicular growth-influencing factors are coordinated so that only one follicle is typically chosen for sequential terminal maturation and ovulation. During mid-gestation, approximately 6–7 million ovarian follicles are at their peak, dropping to about 2–3 million primordial follicles at birth. Luteinizing hormone (LH) stimulates theca cells to produce androgens, but Follicle-stimulating hormone (FSH) levels and androgen conversion to estradiol are insufficient, failing to select a dominant follicle and, as a result, chronic anovulation [32]. AMH, which is secreted by granulosa cells (GCs), is essential in regulating this balance because it inhibits the transition from primordial to primary follicles [33,34,35,36]. The zona reticularis, theca cell, and the adrenal cortex express many steroidogenic enzymes. The zona reticularis secretes hormones such as androstenedione, dehydroepiandrosterone (DHEA), and DHEA sulfate. It is becoming clear that 11-hydroxyandrostenedione, which is eventually converted into the potent androgen 11-ketotestosterone, is part of the adrenal and possibly theca cell’s steroidogenic repertoire [37,38]. Women with PCOS had higher serum levels of the 11-oxygenated androgens: 11-hydroxyandrostenedione, 11-ketoandrostenedione, 11-hydroxytestosterone, and 11-ketotestosterone when compared to control women [39,40].

## 3. PCOS and COVID-19

Because the typical PCOS phenotype is associated with increased IR and hyperandrogenism, impaired glucose tolerance (IGT), type 2 diabetes mellitus (T2DM), and metabolic syndrome (MetS) are significantly more prevalent in women with PCOS [41,42,43]. According to meta-analysis data, women with PCOS have a 4-fold higher T2DM incidence than women without it, independent of and additive to obesity [44,45]. IR is a crucial factor in PCOS pathogenesis, which is linked with a high-risk of MetS. In contrast, its prevalence was highest in phenotypes I and II. Compared to the general population, an increased risk of metabolic abnormalities is found in younger PCOS patients [46]. As a result, the risk factors for severe COVID-19 and the cardio-metabolic diseases common in women with PCOS have a high degree of overlap. This overlap between the unfavorable cardiometabolic profile of PCOS-affected women and the significant risk factors for poorer COVID-19 clinical outcomes suggests that, if exposed to a SARS-CoV-2 infection, this collaborative group of female patients may be at a greater risk than previously thought [8,47,48].

## 4. Microbiome and PCOS

The human gut microbiome contains over 7000 strains, over 1000 species, and from 1013 to 1014 particular microorganisms [49,50]. Bacteria, particularly anaerobes, are the most common micro-organisms in the intestinal microbiome, but viruses, protozoa, archaea, and fungi are also present. *Bacteroidetes* and *Firmicutes* are the two most common bacterial types [51,52,53]. Numerous studies have been conducted in recent years to investigate the relationship between PCOS and changes in gut microbiota [54,55]. The research has shown that PCOS patients are changing and becoming more diverse. The same studies have found that PCOS alters the balance of some bacterial species, such as *Bacteroidetes* and *Firmicutes*, in addition to the overall composition of the microbiome [5]. This change may result in altered short-chain fatty acid production, harming immunity, gut barrier integrity, and metabolism. In terms of the genus *Bacteroides*, Liu et al. discovered that PCOS-afflicted women had a higher prevalence of *Escherichia* and *Shigella* and a gut microbiome composition comparable to that of obese control women [56,57]. The abundance of *Prevotella* species can also be altered; it has been observed that it is increasing in PCOS patients, which may have a negative inflammatory effect on the host [58]. According to another study, an increase in *Prevotellaceae* had an adverse impact because anti-inflammatory metabolites were no longer produced [59]. On the other hand, PCOS patients have significantly lower levels of beneficial bacteria such as *Lactobacilli* and *Bifidobacteria*, which improve immunity and nutrient absorption. Changes in the gut microbiota associated with PCOS are distinct, occasionally contentious, and poorly understood [55,60].

Intestinal flora is a vital “microbial organ” of the human body that is essential to maintaining health. Crosstalk between the insulin receptor’s downstream signaling pathway and the signal transduction of low-grade, persistent inflammation has been observed [61,62]. Endotoxemia, a persistent inflammatory response, short-chain fatty acids, and bile acid metabolism have all been linked to the development of IR. In addition, people with PCOS have been shown to have a significant imbalance in their gut flora [63,64,65,66]. Hence, it can be concluded that gut microbiota mediates systemic low-grade inflammation and IR, affect sex hormone changes and influence the gut-brain axis in PCOS. Intestinal flora, although having external genetic material, influences the expression of host genes, ultimately leading to PCOS. Women with PCOS may be more likely to experience or develop IR due to various causes and mechanisms. As a result, it is crucial to identify further and examine distinct functional bacterial profiles associated with the emergence and progression of PCOS. The hope is that this will open fresh avenues for tailoring treatment. As an added note, more study is required to see if PCOS can be helped by altering the gut microbiome. Finally, it is essential to investigate whether fecal transplant therapies and the usage of probiotics could help treat this problem [5].

## 5. Gut Microbiomes and PCOS

The human gut microbiome is diverse and lively, with a preponderance of bacterial species belonging to *Bacteroidetes*, *Firmicutes*, *Proteobacteria*, *Actinobacteria*, and other microorganisms. Though the gut microbiome is present in the gastrointestinal (GI) tract, its significance extends far beyond regulating the digestive system. The gut microbiome generally supplies the host nutrition and involves various regulatory mechanisms, fat build-up, and energy metabolism. The gut flora structure influences the onset and progression of metabolic and endocrine diseases. Tremellen et al. presented the dysbiosis of gut microbiota (DOGMA) hypothesis that elucidates the etiology of PCOS as: (1) an imbalance in gut flora is caused by obesity or a diet such as low dietary fiber, which disrupts intestinal epithelial cells, thereby increasing the permeability of gut mucosa, (2) IR is caused by the immune system activation that impedes insulin receptor functions due to leakage of lipopolysaccharide (LPS) in the gut, and (3) interfering with follicular growth HI or IR promotes testosterone [67]. Studies have attempted to mark a connection between the gut microbiome and PCOS. Serum dimethylamine was increased in PCOS patients indicating high gut microbiome activity [68]. It was confirmed that by affecting intestinal wall permeability, the gut microbiome was involved in IR and menstrual disorders of PCOS patients [58,69,70].

### 5.1. Gut Microbiome Changes

The variations in gut microbiota between healthy controls and PCOS patients underlie their susceptibility to numerous diseases. A study discovered no significant changes in lower taxa and a reduction in the number of bacteria from *Bacteroidetes* and *Tenericutes*, not more than 1% [71,72]. Alteration in *Bacteroidetes* and *Firmicutes* [73,74], along with other microbial compositions, leads to the altered production of short-chain fatty acids (SCFAs), which in turn has an impact on gut barrier integrity, immunity, and metabolism [58]. In another study by Liu et al. in 2017, the overall gut microbiome content was similar to obesity control with increased *Escherichia* and *Shigella* in PCOS patients [73]. *Lactobacilli* and *Bifidobacterium* are seen as reduced in PCOS patients promoting nutrient absorption and enhancing immunity [54]. It was reported that *Bacteroides vulgatus* was abundant along with reduced levels of glycodeoxycholic and tauroursodeoxycholic acid, leading to interleukin-22 (IL-22) changes [75]. It has been discovered that the increase in *Prevotella species* has a detrimental inflammatory effect on the host [76]. In contradiction, another study indicated a loss in anti-inflammatory metabolites due to the decrease in *Prevotella species* [77]; these changes are demonstrated in Table 1. The changes in the gut microbiota in PCOS women are not fully understood and are sometimes contentious.

### 5.2. IR concerning Gut Microbiomes

IR is a prevalent PCOS problem that severely impacts a patient’s metabolism. It is reported in many human and animal studies that IR was associated with gut microbiome diversity and composition in PCOS women. A study reports that a decrease in *Prevotellaceae* and an increase in *Bacteroides* in PCOS adults when compared to healthy controls was influenced by IR [77]. The development of IR and increased body fat were found after healthy intestinal flora transplantation in germ-free mice. By rousing the immune system, chronic low-grade inflammation (CLGI) is caused by the disturbance of the gut microbiome and increases intestinal permeability [78]. Pro-inflammatory cytokines cause IR disrupting the insulin receptor function. The rise in insulin and blood glucose level was due to heightened intestinal permeability and subsequent entry of LPS in humans and mice [78,79]. Peptide YY (PYY) and ghrelin are involved in IR and intestinal microbiome association and also have a negative connection with body mass index (BMI) [74,80]. Though gut flora alters PYY and ghrelin levels, causing insulin resistance, few studies have observed significant differences [81].

### 5.3. Sexual Hormones concerning the Gut Microbiome

Sex hormones and gut microbiome are related; *Prevotella* was found more abundant in men than women with positive testosterone levels and abnormal estradiol concentrations [82]. In the study with the letrozole-induced PCOS rat model, compared to healthy controls, *Lactobacillus*, *Ruminococcus*, and *Clostridium* species decreased in PCOS rat models, and increased *Prevotella species* were noted [83]. In other studies, *Clostridia*, *Desulfovibrio*, and *Methanobrevibacter* were found less [84,85,86]. The effect of castration on the gut microbiome caused by sex differences was evaluated in the after stages on rats [87,88]. Sex hormones determine the alteration of β-glucuronidase activity and intestinal or systemic immunity by activating the GI tract receptors. It was hypothesized that a relationship between intestinal microbiome changes and HA is caused by PCOS [89]. The gut microbiome was compared between healthy controls and PCOS patients, and the bacterial species’ α and β diversity were reduced, as mentioned in the earlier reports [73,74]. A study showed a correlation between HA and microbiome diversity using single and multiple linear regression analyses [74]. HA may affect the microbiota structure, altering intestinal permeability and triggering the IR process. Furthermore, androgen secretion can enhance visceral adipose tissue decomposition, with a fatty acid increase, further aggravating IR levels promoting PCOS occurrence and development [78].

### 5.4. Mechanism of Bile Acid and IL-22

The clinical significance of IL-22 in PCOS-induced mouse models has been explored; however, the results are still unknown. The role of IL-22 in HA regulation, ovary morphology, IR, and the estrous cycle was studied in the PCOS-induced mice model [90]. The researchers postulated that IL-22 increased adipose tissue browning resulting in altered ovarian function and insulin sensitivity in PCOS women affected by HA [90]. A decrease in IL-22 levels in PCOS patients was found, which reduced glycodeoxycholic acid (GDCA) and tauroursodeoxycholic acid (TUDCA) levels after transplanting to mice fecal microbiota, alterations in bile acid metabolism, infertility, and deterioration of ovaries [75]. These findings imply that administering exogenous IL-22 and altering the gut microbiome may be a viable treatment for PCOS [91]. They also discovered abundant bile salt hydrolase genes producing bile salt hydrolases in *Bacteroides vulgatus*, with a decrease in GDCA and TUDCA levels in PCOS groups. The gut microbiome alterations change the cytokines intestinal immune cells produce. After oral gavage with *Bacteroides vulgatus* or fecal treatment IL-22, mRNA levels were reduced in mice models obtained from PCOS patients. The GATA binding protein 3 (GATA3) mRNA level increased when administered with GDCA, which was essential for IL-22 production. Through the GATA3 signaling pathway, bile acids may stimulate the production of IL-22 synthesis [92].

### 5.5. SCFAs, Gut Hormones, and the Hypothalamic Pituitary Gonadal (HPG) Axis

SCFAs are small molecules such as acetate, butyrate, and propionate that regulate the host’s gut immunity and energy metabolism [93]. These SCFAs have reduced levels observed in PCOS women than in normal controls [94]. Some microbes, such as *Akkermansia*, *Blautia*, and *Roseburia,* are found in reduced amounts leading to less biosynthesis of SCFAs. Because of decreased SCFA synthesis, females with PCOS had lower levels of ghrelin and PYY gut hormones. SCFAs from human colonic cells are claimed to boost the production of glucagon-like peptide 1 (GLP-1) and PYY [95]. G-protein-coupled receptors (GPCRs) determine this production in the intestinal epithelium expressed by L-cells [96]. By influencing the HPG axis, gut hormones can impact PCOS. In the *in vitro* process, the HPG axis GLP-1 regulates the gonadotropin hormone that affects testosterone and LH. In rats, PYY is said to elevate FSH and LH secretion, while in humans, ghrelin shows lower secretion [97]. The variations in gut hormones are associated with changes in SCFA metabolisms mediated by intestinal flora, causing modifications in androgen levels and gonadotropin via the HPG axis to influence PCOS characteristics. More research is required to determine the precise stimulatory and inhibitory functions and which species of the intestinal microbiota influence gut hormone levels by SCFA production [89].

### 5.6. Mechanism of Intestinal Permeability-LPS

Intestinal microbiota changes can lead to a low gut barrier. In PCOS females, heightened zonulin levels were identified [69,72]. With increased permeability, Gram-negative bacteria cell walls can release LPS into the body. LPS binds to receptors causing inflammation that increases the pro-inflammatory cytokines and induces the nuclear factor-kappa B pathway (NF-kB) [98]. In follicular fluid and serum levels, the TNFα and IL-6 are increased by inflammation activity [99]. Testosterone levels rise as LH androgens elevate due to IR caused by inflammation in the ovaries, which limits the synthesis of sex hormones [100]. High androgen concentrations promote IR, resulting in belly and stomach obesity [101]. These findings imply that the intestinal microbiota affects PCOS individuals by promoting IR and enhancing gut permeability, causing endotoxemia and high androgen secretion leading to inflammation.

## 6. Metabolites Contributing to the Development of PCOS

The human gut microbiome comprises approximately 10^14^ microorganisms that produce metabolites and interact with the reproductive system [102,103]. Many studies examined the dysbiosis of the gut microbiome and metabolite changes in PCOS patients, including bile acids, SCFAs, ceramides, and trimethylamine N-oxide (TMAO). Bile acids are cholesterol-derived and re-metabolized by intestinal bacteria, while ceramides can synthesize inside the body tissues [75,104]. SCFAs and TMAO is found in food and react with intestinal bacteria as they cannot be synthesized in the body [83,105]. 

### 6.1. Microbiota Dysbiosis of PCOS

Several studies explored gut microbiome dysbiosis in mouse models and PCOS patients in the past few years. In the letrozole-induced PCOS mice model, hyperandrogenaemia modifies the gut microbiota regardless of the diet intake [106]. Testosterone and metabolic factors are positively related to *Streptococcus*, *Escherichia*, *Shigella*, and *Bacteroides*, while ghrelin is negatively related to *Escherichia*, *Bacteroides*, *Shigella*, *Streptococcus*, and *Akkermansia*. Additionally, it was discovered in their study that there is an increase in LPS-producing bacteria and decreased levels of Akkermansia [73]. Comparably, another study discovered that *Bacteroides fragilis, Parabacteroides merdae*, *Escherichia*, and *Shigella* enhanced metagenomic analysis. The study also claimed that the microbiome might affect gut permeability and cause barrier dysfunction as some bacteria produce LPS and reactive oxygen species (ROS) [107]. A study identified no differences in the bacterial community of normal and high BMI PCOS patients and, due to the bacterial stress response, they lead to an FK506-binding protein 5 DNA-methylated stress condition [108]. There is a difference in bacterial diversity in PCOS patients with IR [89]. Mammadova et al. said that lean PCOS patients have a similar intestinal microbiome to controls in bacterial diversity and richness [109]. The microbial composition of the lower genital tract in the cervical and vaginal canal of 97 reproductive women showed an abundance content of *Chlamydia trachomatis*, *Prevotella*, *Gardnerella vaginalis*, and a decrease in *Lactobacillus species* between PCOS patients and healthy controls [110]. Numerous studies revealed a close relationship in the female reproductive tract between *Lactobacillus species* and the outset of pre-term labor, infertility, vaginosis, abortion, and other detrimental pregnancy outcomes [111,112,113]. Bacterial vaginosis can be arduous to treat because patients are more prone to infections associated with *Prevotella* and *Prevotella* species [112,114,115]. Another study stated that the *Mycoplasma genus* was abundant in PCOS patients and could serve as a potential biomarker for PCOS screening [116].

### 6.2. Bile Acids

Bile acids emulsify fats and help digestion in the GI-tract, and bacteria are necessary for bile acid transformation. Cholic acid and chenodeoxycholic acid from cholesterol in the liver link glucine and taurine, forming primary conjugated bile acid and are carried to the intestine [117]. Primary bile acids are changed to deoxycholic and lithocholic secondary bile acids during interaction with intestinal flora [118]. The G-protein-coupled bile acid receptor (TGR5), vitamin D receptor (VDR), and farnesoid X receptor (FXR) are regulated by the gut microbiome and involved in bile acid metabolism, synthesis, and reabsorption. The gut microbiome inhibits cholesterol 7 α-hydroxylase (CYP7A1) through triglycerides and regulates fat synthesis [119]. FXR in human and mice intestinal tracts promotes fibroblast growth factor 15 (FGF15) and FGF19 expression by inhibiting cytochrome P450 7A1 and cytochrome P450 8B1 enzymes that participate in bile acid synthesis [120]. FXR has impacts on different tissues as well. The effect of FXR is inimical in the gut, and its activities may have favorable metabolic effects in the liver, such as alleviating IR and high-fat diet-induced obesity [121]. Another study revealed that some bile acids stimulate glucagon-like peptide-1 (GLP-1) secretion by triggering TGR5 or FXR, thereby offering a therapeutic value for PCOS and reducing blood glucose levels [122,123]. FXR in the gut and ovarian GCs suggest that primary conjugated bile acids lead to HA [124]. Based on preceding investigations in PCOS patients modulating bile acids, the gut microbiome affects lipid and glucose metabolism and inflammatory conditions resulting in various endocrine abnormalities.

### 6.3. Short-Chain Fatty Acids (SCFAs)

SCFAs are linear carboxylic acids with less than six carbon atoms generated by the gut microbiome and derived from dietary fiber in food. Higher concentrations of acetate, butyrate, and propionate through fatty acid receptors such as FFAR2 (GPR43) and FFAR3 (GPR41) play a crucial role in SCFAs metabolism [125]. The FFAR2 and FFAR3 receptors belong to the GPCRs family. Because of alterations in the gut microbiome, SCFAs are said to be different in patients and healthy adults with metabolic disorders. From pancreatic β-cells, SCFAs interact through FFAR2 and FFAR3 receptors in glucose-insulin secretion, inducing the release of peptide hormones that regulate hunger and increase insulin sensitivity [126,127]. The intestinal microbiota is known to synthesize SCFAs in PCOS patients [5]. *Lactobacillus* increased in PCOS patients after four weeks of probiotic treatment, promotes insulin, and boosts the *Faecalibacterium* growth, releasing butyric acid [128]. These findings suggest that SCFAs act on β-cells to enhance insulin production and preserve the intestinal barrier, thereby alleviating PCOS metabolism [50]. SCFAs alleviate T1DM via IL-22 and improve insulin levels by feeding inulin to mice.

### 6.4. Ceramides

Ceramides are lengthy fatty acids required for the metabolism and synthesis of all sphingolipids. IR, T2D, obesity, and reduced glucose tolerance are related to PCOS. Ceramide production and sphingolipid metabolism have inconsistent evidence regarding their role in PCOS. Jiang et al. compared the ceramide concentration in the serum of healthy controls and PCOS patients and found that the concentrations were higher in PCOS patients. A prior study discovered that ceramide levels are higher in obese PCOS women than in lean, healthy adults [129]. Other studies implied higher ceramide concentrations or sphingomyelin from PCOS adult plasma than healthy adults [130]. Another study found a lower ceramide level than normal controls [131]. Gut flora dysbiosis is related to higher ceramide levels that can contribute to metabolic diseases. Johnson et al. revealed that *Bacteroidetes* in the host influence ceramide production and generate sphingolipids [132]. Kayser et al. observed poor glucose metabolism and obesity changes caused by ceramides connected with the intestinal microbiome [133].

### 6.5. Trimethylamine N-Oxide (TMAO)

A derivative of trimethylamine called TMAO, a gut microbiome-derived small organic compound, was formed in the intestine from L-carnitine, choline, and other substances [134]. TMAO is generated from flavin monooxygenase 3 (FMO3) in the liver through trimethylamine absorption [135]. TMAO was linked with T2DM and cardiovascular diseases [134] and a higher risk of IR [136,137]. Further, it was found that TMAO is related to insulin sensitivity and glycemia with the slightest changes and that TMAO with dieting enhances IR [138]. TMAO activates protein kinase R-like endoplasmic reticulum kinase (PERK); however, the primary mechanism is unclear and has received much interest [139]. TMAO is thought to be a probable metabolite in PCOS pathophysiology. The authors discovered that TMAO and its intermediates are higher in PCOS women than in healthy controls implying that it is related to HA [140]. In PCOS women, intestinal microbiome dysbiosis may be the basis for studying and understanding cardiovascular risk. The modified gut microbiome causes heightened cardiovascular risk because of its pro-atherosclerotic impact and is responsible for elevated TMAO levels in serum [141].

## 7. The Metabolic Perspective of PCOS

### 7.1. Interconnection between Insulin and PCOS

Increased androstenedione (ASD), DHEA, testosterone levels, and decreased sex hormone-binding globulin (SHBG) lead to HI in women [142]. Endothelial dysfunction resistance and insulin are related to PCOS. An inflammatory mediator tumor necrosis factor (TNF) in PCOS females can hasten the IR. IR in endothelial artery cells is caused by elevated endothelin 1 level (ET-1) and reduction in nitric oxide [43]. Insulin with increased androgen synthesis likely enhances adrenal cortex sensitivity to adrenocorticotropic hormone (ACTH) activation in PCOS females. Androgen increases with heightened insulin related to reduced SHBG levels. It suppresses SHBG synthesis indirectly through the glucose and fructose-stimulated inhibition of hepatocyte nuclear factor 4 alpha (HNF-4α) [143]. Insulin increases progesterone and testosterone levels in PCOS women. The reports suggest that IR is associated with reduced SHBG and a rise in testosterone [144].

### 7.2. Obesity-Culprit in the Pathogenesis of PCOS

Females with PCOS develop dysglycemia, with 10% having T2DM and 30 to 40% having low glucose tolerance and MetS [145]. It is said that high androgen secretion promotes fat deposition and induces the ovary and adrenal to produce androgen in excess. Menstrual abnormalities and anovulation appear more intense in overweight PCOS women than in normal women. IR and HA are triggered by fat deposition and adipose malfunction, increasing androgen secretion in the ovary. Steroid synthesis is modified by obesity through aromatase, triggering estrogen in adipose tissues. 

An altered estrogen level increases the LH and decreases the FSH, resulting in hyperplasia of GC and theca cells. These further increase the androgen production in PCOS-obese women and cause HA and hyperoestrogenaemia [143]. In a recent study, a link between obesity and ovulatory failure in PCOS patients was explored in mice, and increased IL-10 levels were found to weaken folliculogenesis [146]. Lerner et al. indicated that high androgens impede adipogenesis in brown adipose tissue by decreasing mitochondrial respiration and attenuating the initiation of thermogenesis [147]. Zhou et al. uncovered the CHRDL1 gene by inhibiting bone morphogenetic protein 4 (BMP4) or by regulating insulin-like growth factor 1 (IGF-1) in obesity-related PCOS [148].

### 7.3. Inflammation

Signaling proteins known as cytokines are small proteins produced by special immune cells that strongly influence other cells [149]. Cytokines are released in ovaries by oocytes, follicular cells, and leukocytes. These act as both autocrine and paracrine regulators in the ovary. The findings suggest that cytokines are involved in embryonic and reproductive development [150]. Specific pro-inflammatory cytokines in Peri-ovarian adipose tissue (POAT) of DHEA are enhanced in the PCOS rat model [151]. PCOS females have altered cytokine levels with obesity, diabetes, and IR-linking inflammation. In the ovary, macrophages in adipose tissue generate an inflammatory response by secreting TNFα and IL-6, and cytokines [152]. In a study, the adipocytokines levels were altered, leading to obesity but not PCOS. Itis due to increased leptin mRNA expression and plasma levels. Adiponectin decreases, indicating elevated IL-6 levels [153]. Looking at the interaction of inflammatory markers and PCOS helps explore more.

### 7.4. An Imbalance between Inflammatory Markers

An inflammatory marker is a biomarker used clinically to detect the inflammation caused by various diseases. These are said to influence female reproductive health as well. For the ovary to function effectively, inflammatory markers are essential. In general, it is known that pro-inflammatory and anti-inflammatory cytokines result in ovarian malfunction. In recent years, inflammatory markers like TNFα, IL-6, IL-8, IL-10, IL-18, and C-reactive proteins (CRPs) have been related to PCOS (Table 2).

#### 7.4.1. Tumor Necrosis Factor-α (TNFα)

Activated macrophages, neutrocytes, and fibroblasts in ovaries produce TNFα. In humans, TNFα is found in follicular fluid during ovulation [104], inhibiting the insulin receptor tyrosine kinase phosphorylation. TNFα is associated with obesity-related IR. Reducing the glucose transporter type-4 (GLUT-4) activity, this cytokine affects the glucose mechanism [143]. Another study reveals a correlation between TNFα and HA. PCOS is aggravated by obesity, which in turn leads to an inflammatory state [154].

#### 7.4.2. IL-6

IL-6 is a pleiotropic signaling inflammatory marker present in all cell types [161]. This cytokine modulates corpus luteum activity and influences the development of the fetus and sex hormone production [162]. It is said that interferon-γ (IF-γ), IL-1, and TNF-α induce IL-6. Increased IL-6 concentrations in serum were observed in PCOS patients causing IR. It has been explored that androgens elicit immune responses in obesity-related PCOS females [163]. A study explained that IL-6 poses adverse effects and is higher in PCOS women with infertility than in healthy controls [164]. Raised IL-6 levels are related to androgens and IR but not BMI [155]. IL-6 mRNA levels increased in PCOS patients, and, as treated with an anti-inflammatory agent such as resveratrol, their expression seemed to decrease, reducing the diabetes risk in patients [156].

#### 7.4.3. IL-8

It is an inflammatory cytokine that helps in ovulation and follicular maturation. It is revealed that vascularisation is developed due to IL-8 in follicular development. A study described that IL-8 is related to metastasis, angiogenesis, and melanoma [165]. IL-8 expression in a clinical trial initially increased, but when treated with pioglitazone and metformin, their expression levels decreased [166]. In comparison with obese PCOS and Non-PCOS patients, the IL-8 levels from GCs increased with increases in BMI, but IL-8 levels decreased in the serum [143,157].

#### 7.4.4. IL-10

IL-10 is an immunosuppressant that plays a vital role in the body’s defense mechanism. It is said that IL-10 is associated with Th2 cells blocking Th1 cell activity. Th1 cells decrease and maintain pregnancy by synthesizing progesterone and corpus luteum production [143]. Lower IL-10 levels are related to obesity and metabolic disorders [167]. Reduced IL-10 leads to oxidative stress in PCOS, leading to inflammation and androgen synthesis [158]. Plasma IL-10 was decreased in patients with PCOS syndrome [168]. However, Sylus et al. noticed that clomiphene citrate regulates IL-10 and increases pregnancy and ovulation rates in women with PCOS symptoms [169].

#### 7.4.5. IL-18

It is a signaling cytokine molecule that activates a cascade of inflammatory cytokines such as IL-1β, IL-2, IL-6, and TNFα receptors. Caspase-1 cleaves pro-IL-18, then IL-18 activates inflammation cytokines and stimulates NF-kB. This molecule is essential for atresia, steroidogenesis, and ovary maturation [143]. A study indicated that elevated IL-18 levels affect the ovary, which leads to folliculogenesis disruption [159]. It is exciting to notice that IL-18 is an essential key element that anticipates deaths due to cardiovascular diseases. People with obesity symptoms have IL-18 more in their serum concentration. However, the IL-18 was reduced in normal-weight cases [143]. Similarly, the IL-18 level was more in the endometrium of PCOS patients with obese conditions when compared with normal weight [170]. Recently, some studies revealed a strong relationship between serum IL-18 and patients with PCOS conditions [171,172].

#### 7.4.6. C-Reactive Protein (CRP)

CRP is a protein produced by the blood in the liver [173]. This protein is controlled by inflammation-producing components, viz. TNF-α and IL-6. It is believed to be a decisive predictive factor for inflammation. The CRP is mainly discharged into the bloodstream through tissue damage and inflammation. Higher concentrations of CRP were observed in PCOS symptoms in obese and nonobese people, which is attributed to cardiovascular risk in PCOS patients [174]. A research explained that elevated levels of CRP were found in women with PCOS irrespective of their obesity condition [175]. Another study revealed that obese females with PCOS with high CRP levels are highly vulnerable to elevated cholesterol or lipids in the blood [176]. It was observed that there are higher levels of CRP drop-off endothelial function in PCOS patients [177].

CRP is a protein produced during the acute phase of the immune response in response to pro-inflammatory chemicals such as TNF-α and IL-6. It is the most important indicator of long-term outcomes in inflammatory diseases [178]. When cells are damaged or inflammation occurs, CRP is released into the bloodstream. Increased CRP predicts systemic inflammation [179,180]. PCOS patients with elevated C-reactive protein levels may be at a greater risk for developing T2D and cardiovascular disease [181]. High levels of CRP are inflammatory and increase the risk of T2D in women with PCOS [182,183]. Research on PCOS and persistent low-grade inflammation has concentrated mainly on CRP levels. This protein is generated in the liver during the acute phase in response to stimulation by IL-6 and TNF-α. Adipose tissue is another source of CRP production. There is mounting proof that CRP is a vital predictor of the onset of cardiovascular illnesses and may serve as a measure of the intravascular inflammatory process. Kelly et al. [179] presented the first evidence showing increased CRP in PCOS patients by comparing 17 PCOS patients with 14 healthy controls. Even after controlling for BMI and age, they still detected a statistically significant increase in serum CRP among the study group [184,185,186,187].

## 8. Vaginal Microbiota

There are significant variations in the vaginal microbiota between pre-pubertal and postmenopausal women owing to the lower genital tract microbiome being altered by age, sex hormone levels, irregular menstruation, abnormal hormone levels, and living practices [188]. Due to hydrogen peroxide plus, lactic acid released by *Lactobacillus sp.*, the vagina is generally acidic with a lower pH. Numerous bacteria exist in vaginal secretions, and the host provides resources for their development and growth. Many investigations have shown that the microbiome makeup alters and that dysbiosis occurs in PCOS in both animal models and individual females [58]. 

The cause of PCOS is still uncertain; however, various variables have been recognized as causing a hormonal and metabolic imbalance, which can contribute to the development of this condition [189]. Furthermore, several studies in the past few years have focused on exploring the significance of vaginal microbiota and its role in PCOS [116]. The microbiome in the vagina is a distinct flora mainly represented by *Lactobacillus spp.* in a healthy person. Other bacteria constitute under 10% of the total microbial flora and are highly present in low numbers [190]. Depending on the amount of Lactobacillus in samples collected from the vagina, the vaginal microbiome may be differentiated into Lactobacillus dominant and non-Lactobacillus dominant groups. The makeup of the two microbial floras varies significantly, which may affect the diversity of the analyses [191]. 

There was a clear relationship between flora composition and FSH in PCOS patients. Several anaerobic bacteria and facultative anaerobes were shown to be strongly inversely connected with FSH levels, while Lactobacillus was found to be directly associated with FSH concentrations. Streptococcus is a prevalent bacterium causing infections. Group B Streptococcus, out of the Streptococcus species, is one of the most common species causing prenatal infection in women and has been reported abundantly in direct association with age and estradiol [192]. Streptococcus may influence PCOS pathology by influencing the endocrine system in the female reproductive organs [191]. Increased levels of *Mycoplasma* genus associated with PCOS were also reported in a recent case-control study [116]. Another study has talked about the involvement of sex hormones. Sex hormones such as estradiol and progesterone were likely responsible for regulating vaginal microbiota, and the inflow of leukocytes was adversely linked with microbe colonization in the vagina [92].

## 9. Metabolomic Insight into PCOS

The metabolome reflects the genetic phenotype and the changes caused by other variables, including age, nutrition, or physical exercise. Metabolomics allows monitoring an organism’s condition and offers information on the molecules generated due to several biochemical activities [193]. The use of metabolomics provides potential insight into PCOS research works. It is a crucial and developing method for discovering new metabolites that may be potential biomarkers for various metabolic and endocrine problems [103]. Plasma and urine are the most often employed matrices in metabolomic research related to PCOS. Urine and serum samples are also more commonly used since they are easy to collect and prepare. Lately, a novel alternative matrix, which is follicular fluid, has been introduced, particularly regarding oocyte maturation and quality [194]. Several limitations are also associated with metabolomic studies in PCOS. Inter-individual variability has considerable difficulty in research that uses human matrices, especially in women, where the range of measured metabolites might be associated with varying hormone levels during the menstrual cycle [195]. The metabolic research studies were conducted on a limited number of female individuals with mainly tentatively determined metabolites. There might also be issues with the performance of the analyzers, which can occasionally provide false positive readings. Variations in sample preparation substantially influence the outcome as well [196].

## 10. MetS and PCOS

MetS is a collection of dysfunctional metabolic characteristics that include obesity, IR, hyperglycemia due to poor glucose metabolism, elevated blood pressure, and abnormal blood lipid levels. The incidence of MetS in women with PCOS is as high as 33%, and it is linked to long-term effects, including T2D, sleep apnea, psychiatric issues, and cardiovascular problems [197,198]. A study has shown that the incidence of metabolic disorders in PCOS is lesser in Italian females than in women from the United States, indicating that hereditary variables, but mostly changes in lifestyle and nutrition, significantly impact the frequency of metabolic disorders in PCOS women [199]. Women suffering from PCOS, regardless of race or ethnicity, develop metabolic syndromes at a young age. HI, a key component in the pathophysiology of PCOS, appears to represent a fundamental connection linking PCOS with MetS [200].

## 11. Serum Metabolomics in PCOS

Metabolomics is a fast-developing area of research that promises to advance understanding the biological significance of metabolic alterations from a single cell to the entire organism. There are two ways to research metabolomics: untargeted, which assesses the number of metabolites in samples without previous knowledge of these chemicals, and another one is targeted metabolomics. Blood has been the subject of comprehensive biochemical evaluation for more than 70 years since it is an essential and readily attainable biological fluid [201,202]. The serum metabolome is a pathologic readout in whole-body metabolism that may also function as a biochemical biomarker [203]. Pseudo-targeted metabolomics, often quasi-targeted or broad-targeted metabolomics, is a complete information-rich technique. It combines the benefits of untargeted and targeted analysis and introduces a new scale for investigating possible biomarkers for clinical diagnosis and medication mechanisms [204].

### Targeted and Untargeted Metabolomics

Targeted metabolomics measures defined sets of chemically determined and biochemically characterized metabolites. Analysis can be done quantitatively or semi-quantitatively using internal standards [205]. Using a targeted strategy, evaluating hundreds of metabolites from several chemical classes is feasible. This method will help identify and quantify various small-molecule metabolites in biological materials, such as amino acids, lipids, organic acids, and nucleotides. A recent study focused on broad-spectrum targeted metabolomics analysis to examine the blood concentrations of metabolites in PCOS individuals and compare them to healthy controls with different BMIs [206]. 

The untargeted metabolomic technique, also called metabolic fingerprinting, focuses on identifying and quantifying as many low-molecular-weight molecules in analyzed samples. This method is often used to discover metabolic profiles and markers and gain fresh insights into the processes behind the pathophysiology of human disorders such as PCOS [207]. In a recent investigation, the untargeted metabolomics of patients affected with PCOS discovered about 146 significantly various metabolites in the serum [208]. The findings indicate that untargeted metabolomics is a potential tool for investigating metabolic anomalies in PCOS patients, which might be valuable for PCOS mechanism research and provide a strong promise for PCOS diagnosis [209].

## 12. Metabolic Dysfunction in PCOS

### 12.1. IR in PCOS

PCOS is characterized using IR [210]. Ovulatory abnormalities and the continued progression of endometrial dysfunction are both direct results of this condition and contribute to a woman’s inability to conceive a child. The long-term adverse consequences of IR on PCOS patients’ metabolisms have been documented [211,212,213]. The overproduction of insulin can stimulate the secretion of LH [214] and androgen secretion by the ovary and the adrenal gland [35]. It can also block the production of hepatic SHBG and raise the body’s free testosterone levels. The overproduction of this hormone has been linked to hirsutism, acne, alopecia, and the suppression of ovarian follicle growth and development [215,216]. 

Furthermore, insulin can significantly affect ovarian follicle growth and hormone levels through insulin receptors in follicle membrane cells. In addition, insulin can raise the ovary’s free IGF by increasing the activity of the ovarian IGF-1 receptor [217]. Women with PCOS are more likely to develop MetS, and IR is a critical player in the route of putative pathophysiological processes accountable for this [210,218]. An elevation in serine phosphorylation leads to abnormalities in insulin signaling and is a significant reason for IR in women with PCOS. Furthermore, adipocyte, skeletal muscle, and ovarian metabolic pathways are all impacted by impairments in insulin receptor and insulin receptor substrate-1 tyrosine phosphorylation [219]. 

Diabetes, IR, and persistent inflammation have all been linked to oxidative stress in women with PCOS [147]. Hyperglycemia and elevated amounts of free fatty acids cause a rise in ROS generation, increasing oxidative stress. TNF-α is produced by polymorphonuclear cells in response to hyperglycemia, which contributes to inflammation. Researchers found that in lean, healthy women of reproductive age with hyperglycemia, surplus androgen increased leukocyte ROS production, p47phox gene expression, and MDA formation [220]. Both obesity and IR are considered central mechanisms in developing PCOS in obese and lean individuals. According to research by Lee et al., a BMI of > 27 or higher is at a high risk of having diabetes in obese PCOS women [221].

### 12.2. Non-Alcoholic Fatty Liver Disease (NAFLD) and PCOS

**Figure 1:**
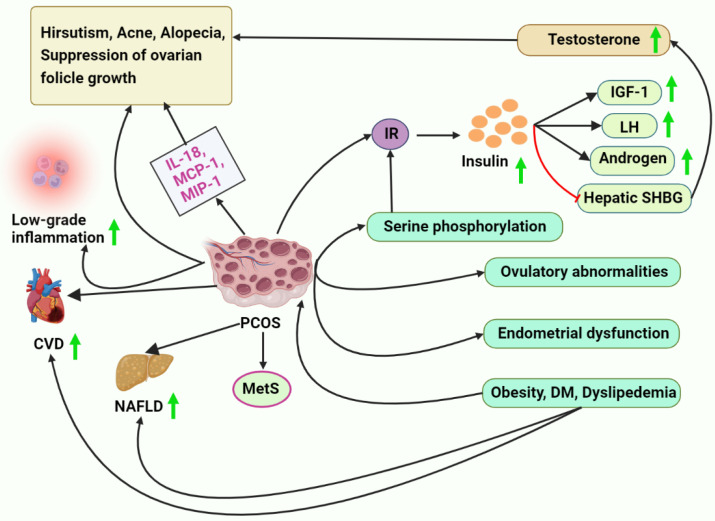
This figure represents various aspects of PCOS. PCOS increases the risk of NAFLD, CVD, and other MetS. Additionally, PCOS leads to serine phosphorylation, ovulatory abnormalities, and endometrial dysfunction. Obesity and dyslipidemia also pose a risk for PCOS.

Women with PCOS have been demonstrated to have a higher likelihood (35–70%) of non-alcoholic fatty liver disease (NAFLD) than healthier women of the same age, BMI, and waist circumference (20–30%) [222,223,224,225,226,227]. Furthermore, NAFLD is typically more serious in individuals with PCOS, and the incidence of PCOS is considerable (50–70%) in those with proven NAFLD [225,226,228]. Among chronic liver diseases, NAFLD is unquestionably the most prevalent. Obesity, DM, dyslipidemia, and MetS are vital contributors to the development of NAFLD. These conditions are also frequently found in PCOS individuals [229,230]. Thus, the link connecting PCOS and MetS is intriguing. Several studies have identified a link between PCOS and NAFLD, with potential HA being an independent risk factor for NAFLD [231,232] (Figure 1).

Furthermore, contrary evidence from other studies suggests that HA is not associated with an increased risk of NAFLD [233]. The MetS and its risk factors, such as IR, obesity, hyperlipidemia, and hypertension, are more common in women with PCOS and NAFLD [234,235]. Despite the mounting evidence linking the two, PCOS and NAFLD share common metabolic comorbidities. Although both NAFLD and androgen excess result from metabolic dysfunction, it is unclear whether the former contributes to the latter. According to a recent meta-analysis, women with PCOS had four times the likelihood of developing NAFLD compared to a healthy control group [236]. According to another meta-analysis, high testosterone levels have been linked to NAFLD in women with PCOS. Women with PCOS have a higher risk of developing NAFLD than women with PCOS who do not have HA, indicating that increased androgen levels promote the occurrence of NAFLD [237]. Possibly contributing significantly to the development of liver illness, the increased production of these androgens fosters an androgen-dependent pro-apoptotic PCOS milieu. Recently, the link between PCOS and NAFLD risk factors such as IR, central obesity, hypertension, and dyslipidemia has been brought to light [238]. The mechanism between PCOS and NAFLD is still unclear because its etiopathogenesis is poorly understood [239].

### 12.3. Cardiovascular Disease (CVD) in PCOS

Clinical studies have shown that PCOS increases a woman’s risk for cardiovascular disease by altering her lipid and glucose metabolism, leading to hypertension and vascular injuries. PCOS may be a cardiovascular risk factor that adversely impairs lifestyle quality due to its early onset [240]. It is essential to recognize the link between PCOS and an increased risk of CVD in the long term and to apply the utmost attention to the possibilities of deploying CVD prevention treatments in these women. Numerous cardiometabolic disorders are often involved in the pathophysiology of PCOS, putting women at an elevated risk for CVD [240,241]. Women with PCOS are at a higher risk of CVD if they have IR and HI [242]. In many studies and meta-analyses, women with PCOS have been at a higher risk for coronary heart disease (CHD) and stroke than women without PCOS [243,244]. Elevated triglyceride (TG), low-density lipoprotein cholesterol (LDL-C), and very low-density lipoprotein cholesterol (VLDL-C) levels, and decreased high-density lipoprotein cholesterol (HDL-C) levels are the most frequent lipid anomalies in women with PCOS [241]. Myocardial infarction (MI) and other cardiovascular diseases can be predicted by lipid abnormalities linked to IR [245]. Obesity is linked to dyslipidemia in women with PCOS because of IR, increased VLDL synthesis, aberrant lipoprotein lipase-mediated lipolysis, and a malfunction in the insulin-signaling pathway caused by the upregulation of the PI3KR1 gene [246]. There is a correlation between IR and hypertriglyceridemia due to the liver’s overproduction of apoB-containing VLDL. Through androgen receptor-mediated IR and the activation of genes for HDL catabolism [246], testosterone causes dyslipidemia in women with PCOS [241]. Traditional CVD biomarker frequency must be determined in PCOS phenotypes. There is some proof that young women with PCOS are more likely to have subclinical atherosclerosis. The evidence implies an elevated incidence of stroke and MI [247], and an elevation in carotid intima-media thickness has been reported [248,249] (Figure 1).

### 12.4. Other Metabolic Consequences of PCOS

PCOS increases the chance of developing metabolic issues. Obesity, IGT, T2DM, dyslipidemia, and hypertension are all classic risk factors for CVD that can coexist with other conditions. Patients with PCOS [250] frequently report worrying about their weight. The prevalence of obesity varies from 50 to 80%, depending on the culture and the people being studied. Women with PCOS tend to be overweight or obese for their whole lives, with the first signs of a change in BMI trajectory appearing as early as the ages of five to fifty-five. PCOS increases a woman’s risk of IGT by a factor of three, regardless of her BMI; the risk is highest among women in Asia and America. There is conflicting evidence on the association between DM and obesity in this population of reproductive age. There is evidence that hypertension and PCOS go hand in hand, although it is not definitive. The longitudinal data show raised blood pressure even in slim women with PCOS [247], but most research fails to show an increased risk of hypertension independent of BMI [251,252].

Endometrial hyperplasia and anovulation-related infertility are both more common in women with PCOS. The risk of endometrial cancer is elevated in premenopausal women with PCOS [253]. A higher rate of mental illness is seen in those with PCOS. Some longitudinal studies show an increased risk of incident depression and anxiety [254], while several cross-sectional studies show an increase in moderate-to-severe depression and anxiety symptoms [255]. In addition, PCOS women are more likely to struggle with an eating disorder [256] and negative body image [257]. Comorbidities and symptoms of PCOS cause life difficult for those with the condition. There is a negative impact of PCOS on the health-related quality of life (QoL) [258,259] that seems to last at least into the late reproductive years [260], and both health professionals and women should know this. There has been little research comparing the risk of dyslipidemia, DM, and MetS in older women with PCOS compared to the results of obesity and IGT. Most information about life after menopause comes from inconclusive cross-sectional studies that include women with a presumptive diagnosis of PCOS [248].

## 13. A CLGI Process in PCOS 

In PCOS, metabolic abnormalities and ovarian dysfunction emerge from a pro-inflammatory state characterized by persistent low-grade inflammation [261]. In response to pro-inflammatory cytokines such as TNF-α and IL- 1 and IL-6, hepatocytes create CRP, a measure of inflammation. An elevated CRP in the blood suggests the existence of CLGI [220]. Higher levels of cytokines and chemokines that promote inflammation are linked to IR, a hallmark of chronic inflammation [179]. Serum oxidative indicators are dramatically higher in patients with PCOS than in normal [184]. Multiple reports have linked PCOS to persistent, low-grade inflammation. Inflammatory indicators or gene markers are elevated in PCOS patients [262].

### 13.1. Metabolic and Inflammatory Markers

The ovary depends heavily on inflammatory indicators for regulation. Ovarian fibroblasts, endothelial cells, neutrophils, and activated macrophages produce the pleiotropic signaling molecule TNF-α [263,264,265,266,267,268]. TNF influences obesity-related IR because this cytokine inhibits insulin receptor tyrosine kinase phosphorylation [269]. Moreover, it influences glucose transfer by decreasing GLUT-4 function. In PCOS patients, this cytokine has a role in the development of IR and obesity [270]. IL-6 [271,272] controls steroid production in the testes, the implantation process, the corpus luteum’s role in pregnancy, and the embryo’s growth and development [273]. Activation of IL-6 is stimulated by pro-inflammatory molecules such as TNF-α, IF-β, and IL-1 [274]. 

IL-8 is an inflammatory cytokine that recruits and activates neutrophils [275]. Ovarian regulation by IL-8 shows promise. Ovulation, follicular growth, and oocyte maturation are all aided by this [276,277,278]. IL-10 is integral to our defense mechanisms because it acts as an immunosuppressant and decreases inflammation [279]. It was first categorized as a Th2 cell because it suppresses Th1 cell function [280]. It is thought that by inhibiting Th1 cell activity, pregnancy can be maintained through the production of progesterone and the development of the corpus luteum [281]. Obesity and MetS are linked to low IL-10 [168,282].

### 13.2. Pro-Inflammatory Cytokines and Chemokines

The health of the ovaries depends on a steady state of inflammatory marker concentrations. According to research, ovarian dysfunction, altered steroidogenesis, and delayed follicular maturation have all been linked to anti-inflammatory and pro-inflammatory cytokine imbalances [283]. Signaling proteins called cytokines are produced by specific immune cells and profoundly affect other cells. Leukocytes, oocytes, and follicular cells in the ovary produce it [284]. PCOS is associated with increased inflammation. T2DM and CVD are inflammation-related [285]. Patients with PCOS have increased levels of cytokines that promote inflammation [286,287]. 

Consequently, cytokines levels are altered in women with PCOS. Increased levels of IL-18, monocyte chemoattractant protein-1 (MCP-1), and macrophage inflammatory protein-1 (MIP-1) have been linked to PCOS [288,289,290]. A member of the IL-1 superfamily, IL-18 is a pro-inflammatory cytokine linked to IR and MetS and is a significant predictor of long-term cardiovascular death. Obese women with HI have considerably greater IL-18 [289,290], although its levels are raised in PCOS patients irrespective of IR and obesity. Serum levels of IL-6 were not different between women with PCOS and controls, according to a meta-analysis by Escobar-Morreale et al. [175]. The findings were comparable to a meta-analysis of CVD risk indicators in women with PCOS conducted by Toulis et al. [291]. TNF-α is another well-known cytokine that mediates IR and is released mainly by visceral adipocytes. However, the two available meta-analyses of the data have not confirmed a correlation between plasma TNF-α levels and PCOS [175,291].

The androgenic hormone DHEAS and the inflammatory cytokines IL-6 and TNF-α are associated with varying degrees of PCOS. Obesity and IR are connected to the pro-inflammatory cytokines TNF-α and IL-6. Both cytokines are produced by adipose tissue, which explains why many women with PCOS have visceral fat accumulations and insulin sensitivity changes [292]. Insulin inhibits the production of inflammatory cytokines and immune mediators [293], causing it to be an anti-inflammatory agent. The anti-inflammatory effects of IL-2 and IL-4 are well-documented in various illnesses, despite the pleiotropic nature of these cytokines. At the same time, there was no statistically significant difference in cytokine levels between groups [294,295]. Toshati et al. (2020) propose that these cytokines are generated as counter-regulators of the sub-clinical and systemic inflammatory process often reported in PCOS women [296].

### 13.3. White Blood Cell Count (WBCs)

The number of WBCs in the blood increases during chronic inflammatory processes. Tola et al. [184] found that CRP was considerably more significant in the PCOS group than in the control group and that the WBCs were dispersed similarly across the two groups. A study by Rudnicka et al. [187] showed that androgens, insulin, and BMI were positively connected with increased WBCs, which were also significantly greater in PCOS than in healthy patients. The leukocyte count in PCOS has also been a predictor by other authors [187,297]. The total testosterone, androstenedione, and DHEAS were also found to correlate well with the WBCs. Leukocytosis may be explained by hyperandrogenemia alone or in conjunction with central obesity and IR. There is still some mystery around the mechanism. According to several studies, androgens have been shown to have therapeutic effects against human leukemia cell lines *in vitro* and *in vivo* [298].

Subclinical inflammation, characterized by increased WBC counts, IL 6, and CRP levels, is a crucial feature of PCOS with or without obesity. The metabolic and cardiovascular problems of PCOS are exacerbated by persistent low-grade inflammation [178,186,299,300], which may be related to the elevated WBC count in PCOS. Oxygen-free radicals are produced when WBCs multiply and become activated, releasing inflammatory mediators, including neutrophilic myeloperoxidase and NADPH oxidase. Atherosclerosis, hypertension, and MetS are all initiated by the oxidation and deformation of LDL in a prooxidant milieu [301]. Neutrophils release the inflammatory enzymes neutral endopeptidase and elastase at the site of endothelial damage [301,302]. CLGI in PCOS has been linked to increased levels of WBCs, the neutrophil-lymphocyte ratio (NLR), and the platelet-lymphocyte ratio (PLR) [303,304,305]. Women with PCOS exhibited statistically significantly increased WBCs compared to their normal-ovulating, non-HA, age-matched peers, primarily due to the additive effect of obesity and IR and not due to PCOS itself [306].

## 14. Therapeutic Opportunities

### 14.1. Probiotics, Prebiotics, and Synbiotics

The benefits of probiotics, prebiotics, and synbiotics in treating PCOS seems appropriate due to their low risks and ease of use. Probiotics and synbiotics are shown to improve hormonal (SHBG and FAI) and inflammatory (MDA and NO) indices in women with PCOS [307]. Women with PCOS who ingested probiotics and synbiotics had higher hs-CRP, NO, TAC, GSH, and MDA levels in their serum [308]. In PCOS patients, probiotic supplementation is reported to lower FPG, LDL-C, TG, and TC levels while having little to no impact on HDL-C and HS-CRP levels [309]. Ahmadi et al. reported a statistically significant reduction in weight and BMI in PCOS patients with probiotic supplementation (*L. casei*, *L. acidophilus*, and *B. bifidum*) for 12 weeks along with beneficial effects on triglycerides (TG), glycemia, and VLDL cholesterol [310]. Women with PCOS were shown to have similar outcomes after taking supplements of *L. casei*, *L. acidophilus*, *L. rhamnosus*, *L. bulgaricus*, *B. breve*, *B. longum*, and *Streptococcus thermophiles* for 8 weeks, which led to a significant drop in serum insulin and plasma glucose levels [311]. Similarly, Rashad et al. discovered that using probiotic supplements (*L. delbruekii* and *L. fermentum*) for 12 weeks significantly decreased Homeostatic Model Assessment of IR (HOMA-IR) levels while also improving lipid profiles [312]. According to Jamilian et al., co-administration of probiotics containing *Bifidobacterium*, *Lactobacillus*, and selenium to PCOS-affected women reduced testosterone and hirsutism levels while also having positive impacts on mental health indicators [313]. Probiotic therapy with *Lactobacillus acidophilus*, *Lactobacillus plantarum*, *Lactobacillus fermentum*, and *Lactobacillus gasseri* has also demonstrated a potential effect in modulating inflammatory processes when performed for 12 weeks on women with PCOS [314].

### 14.2. Fecal Microbiota Transplantation

The disruption of ovarian function and decline in fertility caused by FMT in women with PCOS suggests that altering the gut microbiota may be an effective strategy for treating PCOS [315]. Guo et al. treated rats with FMT in a murine PCOS model. Compared to the untreated control group, the FMT-treated PCOS rats showed significant improvements after 36 days, including lowered androgen levels, a considerable rise in estradiol and estrone, and a normalization of ovarian function. In PCOS rat models, FMT and Lactobacillus transplantation can enhance androgenism and influence insulin function [83]. In contrast to untreated rats, PCOS rat models treated with FMT had better female cycles and decreased androgen production [316].

### 14.3. Short-Term Isoflavone Intervention in PCOS

Genistein, an isoflavone phytoestrogen, has been demonstrated in laboratory tests to directly reduce insulin-stimulated IRS-1 serine phosphorylation in endothelial cells, which inhibits inflammation in an IKKβ/NF-κB-dependent manner [317]. Daidzein restores the TNF-α-mediated decrease in Forkhead box protein 01 (Fox01) despite their structural similarity through separate molecular pathways [318]. Few studies have investigated the connection between isoflavone intake and PCOS in people, despite numerous trials being performed to correlate isoflavones with PCOS in rats. These trials showed that women with PCOS who ingested isoflavones for three to six months saw improvements in their plasma lipid and androgen profiles [319,320,321,322,323,324]. Soy isoflavones were found to decrease the percentage of diestrus days, a measure of monthly irregularity, in an animal study conducted by Rajan et al. [325]. A recent study demonstrated that a brief isoflavone intervention improved fasting blood sugar and insulin sensitivity in PCOS patients but not controls [324]. According to Patisaul et al., feeding rats a soy-based diet during the gestational and postnatal periods can cause irregular menstruation in rats [326]. Furthermore, PCOS clinical research that utilized 36 mg/day of genistein for six months found that the drug’s use had no appreciable impact on menstrual irregularity [327].

### 14.4. Rhizomicrobiomics of Caesalpinia bonducella in PCOS Treatment

The medicinal shrub *Caesalpinia bonducella* (Nicker Bean), which has countless positive effects on human health, has gained recent attention for its remarkable therapeutic efficacy in treating PCOS [328]. The seeds and extracts of this plant are used to treat PCOS, a common hormonal condition that affects one in ten women and can cause complications such as infertility and cancer [261]. It is known for its anti-inflammatory, hypoglycaemic, anti-androgenic, and anti-estrogenic properties [329]. *C. bonducella* phytochemicals are and beneficial against several conditions linked to PCOS [330]. Furanoditerpene-rich *C. bonducella* seeds are suggested for PCOS patients [331]. As a result of the presence of several phytochemicals, ethanolic seed extract of *C. bonduella* (ESECB) could be used to treat PCOS in a way that ameliorates multiple complications, including HI, IR, and HA, and promotes ovulation. Female rats with PCOS induced by mifepristone have shown significant improvements in hormonal (progesterone, testosterone, estradiol, FSH, LH, PRL, and insulin) imbalances following treatment with *C. bonducella*. Another mouse model study showed that *C. bonducella* dramatically lowers metabolic symptoms (hyperglycemia and dyslipidemia) in PCOS complications brought on by mifepristone [332].

### 14.5. Metformin Treatment

The biguanide family drug Metformin, an anti-hyperglycemic medication, has been used extensively in PCOS-positive infertile women [333]. Although it is still an unapproved indication in PCOS, metformin has long been used to manage T2DM and is one of the insulin-sensitizing medications frequently used to treat PCOS [334]. In anovulatory PCOS women, metformin treatment lowers insulin levels, luteinizing hormone (LH) synthesis, and circulating androgen levels [335]. Because metformin increases glucose absorption, less insulin is produced or secreted. The protective effects of this drug include restoring ovulatory cycles, menstrual cycle, and fertility as the abnormal levels of insulin affect the functioning of the hypothalamus-pituitary, and ovary and also glucose use in peripheral tissues [336]. The clinical pregnancy rate with metformin versus placebo was considerably higher, according to a Cochrane analysis of seven RCTs, including 702 women. When metformin was used to induce ovulation in PCOS patients, it was found that both the rate of clinical pregnancy and ovulation increased [337].

### 14.6. Other Glucose-Lowering Medications

A thiazolidinedione, pioglitazone, enhances peripheral glucose uptake, regulates adipogenesis and insulin action, and has a beneficial effect on insulin resistance, hyperandrogenism, and ovulatory dysfunction among women with PCOS [338]. Dipeptidyl peptidase-4 (DPP-4) inhibitors, GLP-1 receptor agonists (GLP-1RAs), and sodium-glucose cotransporter-2 (SGLT2) inhibitors are three more recent classes of glucose-lowering drugs that are emerging as promising treatments for affected women [339]. Dipeptidyl peptidase-4 (DPP-4) inhibitors, commonly referred to as gliptins (e.g., linagliptin, sitagliptin, alogliptin, vildagliptin, and saxagliptin), are oral anti-hyperglycaemic medications for the treatment of type 2 diabetes; they are typically used as a second- or third-line therapy following metformin [340]. Their action is to inhibit DPP-4, an enzyme that breaks down GLP-1 produced internally [341]. There is evidence that incretin hormones, such as GLP-1 and glucose-dependent insulinotropic polypeptide (GIP), stimulate glucose-dependent insulin release, particularly after meals [342] (Table 3).

## 15. Conclusions

Intestinal flora, although external genetic material, influences the expression of host genes, ultimately leading to PCOS. Because it is such a complex system, the ecosystem also plays a role in the onset and progression of PCOS via various interconnected mechanisms [5]. More specifically, the mechanism by which the gut microbiota shifts in various PCOS phenotypes remains unknown and calls for more research. While current research has shed some light on how gut flora may play a role in PCOS, it has not been performed in a way that allows for a complete picture to emerge. The link between an unbalanced intestinal microbiota and PCOS can be better understood if more randomized and controlled trials are conducted. The lack of a clear understanding of how PCOS causes ovulation dysfunction, and IR has hampered the development of effective treatment medications. In addition, various routes and circumstances may affect IR in PCOS women. Therefore, it is critical to identify and investigate the unique functional bacterial profiles associated with PCOS. Hopefully, this will open fresh avenues for tailoring care to each individual. More study is required to ascertain if PCOS may be effectively treated by altering the gut microbiome. Lastly, it is crucial to research whether probiotics and fecal transplant therapy could be helpful in the management of this disorder.

## Figures and Tables

**Table 1:** This table summarizes the changes in PCOS patients due to the presence of microorganisms.

Effect	Microorganism	Changes	References
Increase	*Escherichia* and *Shigella*	Alteration in SCFAs production	[73]
Decrease	*Lactobacilli* and *Bifidobacterium*	Promote nutrition absorption and enhance immunity	[54]
Increase	*Bacteroides vulgatus*	Reduced levels of glycodeoxycholic and tauroursodeoxycholic acid	[75]
Increase	*Prevotella*	Detrimental inflammatory effect on the host	[76]
Decrease	*Prevotella*	Loss in anti-inflammatory metabolites	[77]

**Table 2:** Summarizes the relation between inflammatory markers and PCOS in recent years.

Sample	Effect	Inflammatory Markers	Observation	Reference
Serum and endometrial tissue	Increase	TNFα	PCOS aggravated by obesity condition leads to an inflammatory state	[154]
Serum	Increase	IL-6	Raised IL-6 levels are related to androgens and IR but not to BMI	[155]
Adipose tissue of rat	Increase	IL-6	When treated with resveratrol, IL-6 mRNA expression decreased compared to normal treatment	[156]
Serum and follicular fluid	Increase	IL-8	In GCs with an increase in BMI, increased IL-8 mRNA expression but gradually decreased serum concentration	[157]
Follicular fluid	Decrease	IL-10	Reduced IL-10 leads to oxidative stress in PCOS, which in turn leads to inflammatory and androgen synthesis	[158]
Serum and pooled follicular fluid	Increase	IL-18	Elevated IL-18 levels affect the ovary leading to folliculogenesis disruption	[159]
Serum	Increase	CRP	Obese PCOS females are at IR risk where oxidative stress does not cause obesity	[160]

**Table 3:** This table summarizes the treatment strategies.

Therapeutic Options	Mechanism	Experimental Model	Dosage	Effects	Reference
Probiotics	Regulate sex hormone-related microbes	Sprague Dawley rats	1 × 10^9^ CFU bacteria for 28 days	Reduced MDA and FAI; increased NO and SHBG; increased TAC and GSH levels	[307,343]
Prebiotics	Improve inflammation, antioxidant activity	Humans	20 g for 3 months	Reduced expression of inflammatory and oxidative stress markers	[307,344]
Fecal microbiota transplantation	Restores gut flora alterations	Sprague-Dawley rats	2 × 10^9^ fecal microbiota for 14 days	Decreased androgen level, increased estrogen level; improved ovarian disorder and estrus cycles	[83]
Isoflavone interventions	Have estrogen-modulating, antioxidant, and anti-inflammatory activities	Human	200 mL soy drink; twice a day for 3 days	Improved glucose homeostasis, stool metagenomic pathways, microbial α -diversity	[325]
*Caesalpinia bonducella*	Induces ovulation	Wistar strain adult albino female rats	200–400 mg/kg for 28 days	Ameliorated HI, insulin resistance, and HA	[328]
Metformin treatment	Induces high glucose uptake	Humans	2.55 g/d throughout pregnancy	Reduced gestational diabetes; IR; insulin secretion	[345]
DPP-4 inhibitor: sitagliptin	Increases incretin hormone, GLP1, and GIP	Humans	100 mg/d for 6 weeks	Improved insulin sensitivity; β-cell glucose sensing; lowered oral glucose, glucagon response, and postprandial endogenous glucose release	[346]
Pioglitazone	Improves IR	Humans	30 mg/day for 6 months	Improved endothelial function; decreased cardiovascular risk	[347]
GLP-1 receptor agonists	Reduces BMI; improves IR; decreases abdominal circumferences	Humans	1.2 mg of liraglutide once daily	Improved IR; decreases HA.	[348]

## Data Availability

Data are available from the authors on request (A.V.G.).
